# Comparison of molecular and MALDI-TOF MS identification and antifungal susceptibility of clinical *Fusarium* isolates in Southern China

**DOI:** 10.3389/fmicb.2022.992582

**Published:** 2022-09-20

**Authors:** Penghao Guo, Jianlong Chen, Yiwei Tan, Li Xia, Weizheng Zhang, Xiaojie Li, Yujie Jiang, Ruiying Li, Chunmei Chen, Kang Liao, Yaqin Peng

**Affiliations:** ^1^Department of Clinical Laboratory, The First Affiliated Hospital, Sun Yat-sen University, Guangzhou, China; ^2^Department of Clinical Laboratory, Zhongshan Ophthalmic Center, Sun Yat-Sen University, Guangzhou, China; ^3^Department of Clinical Laboratory, Jieyang People’s Hospital, Jieyang, China; ^4^Department of Clinical Laboratory, Guangzhou No.11 People’s Hospital, Guangzhou, China; ^5^Department of Clinical Laboratory, The Third Affiliated Hospital, Sun Yat-Sen University, Guangzhou, China; ^6^Department of Clinical Laboratory, Central Hospital of Guangdong Nongken, Zhanjiang, China; ^7^Department of Clinical Laboratory, The First Affiliated Hospital, Guangdong Pharmaceutical University, Guangzhou, China; ^8^Department of Clinical Laboratory, The Seventh Affiliated Hospital, Sun Yat-Sen University, Shenzhen, China

**Keywords:** *Fusarium*, humans, sequence analysis, mass spectrometry, microbial sensitivity tests

## Abstract

**Background:**

*Fusarium* species are opportunistic causative agents of superficial and disseminated human infections. Fast and accurate identification and targeted antifungal therapy give help to improve the patients’ prognosis.

**Objectives:**

This study aimed to evaluate the effectiveness of matrix-assisted laser desorption ionisation time of flight mass spectrometry (MALDI-TOF MS) for *Fusarium* identification, and investigate the epidemiology and antifungal susceptibility profiles of clinical *Fusarium* isolates in Southern China.

**Methods:**

There were 95 clinical *Fusarium* isolates identified by DNA sequencing of translation elongation factor 1-alpha (TEF1α) and MALDI-TOF MS, respectively. Antifungal susceptibility testing of isolates was performed by broth microdilution according to the CLSI approved standard M38-A3 document.

**Results:**

Seven species complexes (SC) with 17 *Fusarium* species were identified. The most prevalent SC was the *F. solani* SC (70.5%, 67/95), followed by the *F. fujikuroi* SC (16.8%, 16/95). *F. keratoplasticum* within the *F. solani* SC was the most prevalent species (32.6%, 31/95). There were 91.6% (87/95) of isolates identified by MALDI-TOF MS at the SC level. In most of species, amphotericin B and voriconazole showed lower MICs compared to itraconazole and terbinafine. The *F. solani* SC showed higher MICs to these antifungal agents compared to the other SCs. There were 10.5% (10/95) of strains with high MICs for amphotericin B (≥8 μg/ml), terbinafine (≥32 μg/ml) and itraconazole (≥32 μg/ml) simultaneously, mostly focusing on *F. keratoplasticum* (9/10).

**Conclusion:**

MALDI-TOF MS exhibited good performance on the identification of *Fusarium* strains at the SC level. The *F. solani* SC was the most prevalent clinical SC in Southern China. The MICs varied significantly among different species or SCs to different antifungal agents.

## Introduction

The genus *Fusarium* is an important phytopathogen; only a few species can cause a broad spectrum of human infections (Al-Hatmi et al., 2016b; Van Diepeningen and de Hoog, 2016). Almost 70 *Fusarium* species have been reported as opportunistic human pathogens, with the increasing rates of infection over the past years (Tortorano et al., 2014; Triest et al., 2015). The clinical manifestations of *Fusarium* disease are diverse, depending largely on the immune status of the host and the portal of entry (Tortorano et al., 2014). In immunocompetent patients, *Fusarium* species mainly lead to superficial infections such as keratitis and onychomycosis, while the invasive or disseminated infections tend to affect critically ill and immunosuppressed patients with a high mortality rate (Zhao et al., 2021).

The clinically relevant *Fusarium* species are mainly grouped into six species complexes (SC), including the *F. solani* SC (FSSC), *F. oxysporum* SC (FOSC), *F. fujikuroi* SC (FFSC), *F. dimerum* SC (FDSC), *F. incarnatum-equiseti* SC (FIESC), and *F. chlamydosporum* SC (FCSC; Triest et al., 2015). It has been found that antifungal susceptibility may vary among different species within a single species complex (O’Donnell et al., 2008; Al-Hatmi et al., 2015b; Song et al., 2021), which indicates it is necessary to identify the aetiological agent up to the species level for clinical treatment. In the clinical laboratory, these closely related species are often morphologically indistinguishable. Molecular analysis can provide the gold standard for species identification, while it has the disadvantages of being time-consuming and costly. A rapid, simple, cost-effective, and reproducible tool has received increasingly interest for mold identification, i.e., matrix-assisted laser desorption ionisation time of flight mass spectrometry (MALDI-TOF MS; Triest et al., 2015; Al-Hatmi et al., 2015a; Normand et al., 2021). This approach has been found to enhance the correct identification rate of non-*Aspergillus* filamentous fungi with a 31%–61% increase (Ranque et al., 2014). However, more data are needed for the verification and standardization of *Fusarium* identification due to the potential impacts of different instrument platforms and reference spectrum databases on its performance.

In clinic, amphotericin B and azole drugs, e.g., voriconazole and itraconazole, are commonly used for *Fusarium* infection (Nucci and Anaissie, 2007; Tortorano et al., 2014; Oliveira et al., 2020). Amphotericin B or voriconazole is used for the disseminated infections as first-line drugs (Al-Hatmi et al., 2018). *Fusarium* keratitis is mainly treated with voriconazole and natamycin, and the treatment of onychomycosis should include terbinafine, voriconazole and sometimes itraconazole (Al-Hatmi et al., 2018). However, it has been reported that clinical *Fusaria* have relatively decreased susceptibility to these commonly used antifungal drugs (Taj-Aldeen et al., 2016; Rosa et al., 2019). Different patterns of *in vitro* susceptibility have been found in different *Fusarium* species (Song et al., 2021). Remarkably, since neither clinical breakpoints nor epidemiological cutoff values have been established for *Fusarium* according to Clinical and Laboratory Standards Institute (CLSI) M59-3ed (CLSI, 2020) and EUCAST database,^1^ information on the correlation between minimum inhibitory concentration (MIC) and drug efficacy is not clear. Given that a limited number of studies on *in vitro* susceptibility are available, more data are necessary for the epidemiology and therapy purpose.

Studies on clinical *fusaria* are limited in Asia, especially in Southern China. In this study, we aim to investigate the prevalence characteristics and antifungal susceptibility profiles of clinical *Fusarium* strains collected from eight hospitals in Southern China. And the effectiveness of *Fusarium* identification by MALDI-TOF MS was also investigated.

## Materials and methods

### *Fusarium* strains

Ninety-five clinical *Fusarium* strains were collected from eight hospitals in Southern China between January 2018 and December 2020. These isolates were recovered from corneal scrapings (47.4%, 45/95) and skin secretions (40.0%, 38/95), followed by pus (4.2%, 4/95), blood (4.2%, 4/95), sputum (3.2%, 3/95) and urine (1.0%, 1/95). Duplicated isolates were excluded if they were obtained from the same patient. Given samples were totally collected during routine patient care in this retrospective investigation, the need for informed consent was waived by the institutional review board of the First Affiliated Hospital of Sun Yat-sen University.

The *Fusarium* strains were cultured for 5 days on potato dextrose agar medium at 28°C. All cultures were handled within a class II biological safety cabinet.

### DNA sequencing

A single colony was picked up in a 1.5-ml Eppendorf (EP) tube containing 1.0 ml PBS, with the turbidity adjusted to 1.0 McFarland. DNA extraction was performed using the Yeast Genomic DNA Rapid Extraction Kit (Sangon Biotech, Shanghai, China) according to the manufacturer’s instructions.

The sequence of the translation elongation factor 1-alpha gene (TEF1α) was amplified using the primers EF1 (5′-ATGGGTAAGGARGACAAGAC-3′) and EF2 (5′-GGARGTACCAGTSATCATGTT-3′) as previously described with some modifications (O’Donnell et al., 2008). The PCR amplification was conducted in a 50-μL reaction mixture containing 10 μl 10 × PCR buffer, 5 μl templates, 1 μl forward primer, 1 μl reverse primer, 0.5 μl Taq enzyme, 8 μl dNTP mixture, and 24.5 μl double-distilled water. The PCR amplification condition is as follows: 1 cycle of 95°C 10 min; 40 cycles of 95°C 30 s, 56°C 30 s, 72°C 30 s; 1 cycle of 72°C 10 min. The PCR products were subjected to Sanger sequencing (Sangon Biotech, China). The sequences were identified by BLAST analysis in GenBank^2^ (Da et al., 2021).

### The MALDI-TOF MS analysis

The colonies were picked by sterile swabs in a 1.5-mL EP tube containing 0.9 ml 75% ethanol and 20–30 glass beads, mixed for 2 min. Then the suspension was removed to a new tube for centrifugation at 13,000 rpm for 2 min. The supernatant was removed, and 40 μl freshly prepared 70% formic acid was added to the tube and mixed for 1 min. Then, 40 μl acetonitrile was added to the tube and mixed for 1 min. The tube was centrifuged at 13,000 rpm for 2 min. One μl of supernatant was added on the spot of the target plate, and 1 μl CHCA matrix was added after the 1-μl supernatant dried. After the matrix dried, the target plate was taken to the mass spectrometer’s ionization chamber. The mass spectra of the strains were acquired using a VITEK MS Plus (bioMérieux, France) in IVD mode and analyzed by the IVD knowledge base V3.2 for *Fusarium* identification.

The dendrogram showing taxonomic relationships was carried out using VITEK MS RUO/SARAMIS (bioMérieux, France) according to the manufacturer’s instructions. Firstly, spectra were manually imported to the SARAMIS™ RUO database version 4.17 using the button “import spectra to spectra database.” Then the dendrogram was generated according to the whole spectra. Consensus spectra were analyzed with a single link agglomerative clustering algorithm, applying the relative taxonomy analysis tool of SARAMIS premium software to show the resulting dendrogram with differences and similarities in relative terms (percent matching masses).

For instrument calibration, the *Escherichia coli* strain (ATCC 8739) was applied. And the *Candida glabrata* strain (ATCC MYA-2950) was used as quality control.

### *In vitro* antifungal susceptibility testing

Four commonly antifungal agents (Shanghai Aladdin Bio-Chem Technology Co., Ltd., China), i.e., amphotericin B, voriconazole, itraconazole and terbinafine were included and dissolved in dimethyl sulfoxide to 3.2 mg/ml as stock solutions. The work concentrations of these agents ranged from 0.06 to 32 μg/ml. The broth microdilution was performed according to CLSI M38-A3 method (CLSI, 2017). The colonies were picked up and transferred into a 1.5-ml EP tube containing 1.0 ml PBS, with turbidity adjusted to 0.5 McFarland. The suspensions were then diluted in RPMI 1640 to the desired concentration of 0.4 × 10^4^–5 × 10^4^ CFU/ml by counting on a hemocytometer, 100 μl of which were added in the microdilution plates for 48-h incubation at 35°C. The MICs were defined as the lowest concentration with complete growth inhibition compared to the drug-free growth. MIC_50_ and MIC_90_ values were defined as the lowest concentrations that inhibited the growth of 50% or 90% of the strains. WHONET software version 5.6 was used for determining MIC_50_, MIC_90_, geometric mean (GM) and MIC range.

The strains of *Candida parapsilosis* (ATCC 22019) and *Candida krusei* (ATCC 6258) were used as quality controls.

### Sequence accession numbers

All sequences identified in this study were deposited in GenBank (ON959267–ON959361).

## Results

### Identification

The 95 isolates were identified by DNA sequencing of TEF1α as members of 7 species complexes (SC) with 17 *Fusarium* species (Table 1): FSSC (70.5%, 67/95), FFSC (16.8%, 16/95), FOSC (7.4%, 7/95), FDSC (2.1%, 2/95), one isolate of FIESC, FCSC and *F. nisikadoi* SC (FNSC), respectively. The FSSC was the most prevalent SC, including *F. keratoplasticum* (32.6%, 31/95), *F. falciforme* (20.0%, 19/95), *F. solani sensu stricto* (6.3%, 6/95), *F. ambrosium* (5.3%, 5/95), *F. petroliphilum* (4.2%, 4/95) and *F. lichenicola* (2.1%, 2/95). The FFSC included *F. proliferatum* (7.4%, 7/95), *F. sacchari* (3.2%, 3/95), *F. concentricum* (3.2%, 3/95), *F. verticillioides* (2.1%, 2/95) and *F. napiforme* (1.1%, 1/95). The FOSC included *F. oxysporum* (6.3%, 6/95) and one isolate of *F. acutatum.*

**Table 1:** Comparison of identification results of 95 clinical *Fusarium* strains using DNA sequencing of TEF1α and MALDI-ToF MS methods.

DNA sequencing (No.)	MALDI-TOF MS, No.					
	SC level			Species level		
	Correct	Unidentified	Misidentified	Correct	Unidentified	Misidentified
***F. solani*** **SC (67)**						
*F. keratoplasticum* (31)	31	0	0	0	31	0
*F. falciforme* (19)	19	0	0	0	19	0
*F. solani sensu stricto* (6)	6	0	0	0	6	0
*F. ambrosium* (5)	5	0	0	0	5	0
*F. petroliphilum* (4)	4	0	0	0	4	0
*F. lichenicola* (2)	2	0	0	0	2	0
***F. fujikuroi*** **SC (16)**						
*F. proliferatum* (7)	7	0	0	7	0	0
*F. sacchari* (3)	2	1	0	0	1	2
*F. concentricum* (3)	1	2	0	0	2	1
*F. verticillioides* (2)	2	0	0	2	0	0
*F. napiforme* (1)	0	1	0	0	1	0
***F. oxysporum*** **SC (7)**						
*F. oxysporum* (6)	6	0	0	0	6	0
*F. acutatum* (1)	0	1	0	0	1	0
***F. dimerum*** **SC (2)**						
*F. dimerum* (2)	2	0	0	2	0	0
***F. chlamydosporum*** **SC (1)**						
*F. chlamydosporum* (1)	0	1	0	0	1	0
***F. incarnatum-equiseti*** **SC (1)**						
*F. incarnatum* (1)	0	1	0	0	1	0
***F. nisikadoi*** **SC (1)**						
*F. commune* (1)	0	1	0	0	1	0
All isolates	87	8	0	11	81	3

TEF1α, translation elongation factor 1-alpha; MALDI-ToF MS, matrix-assisted laser desorption ionisation time of flight mass spectrometry; SC, species complex.

**Figure 1:**
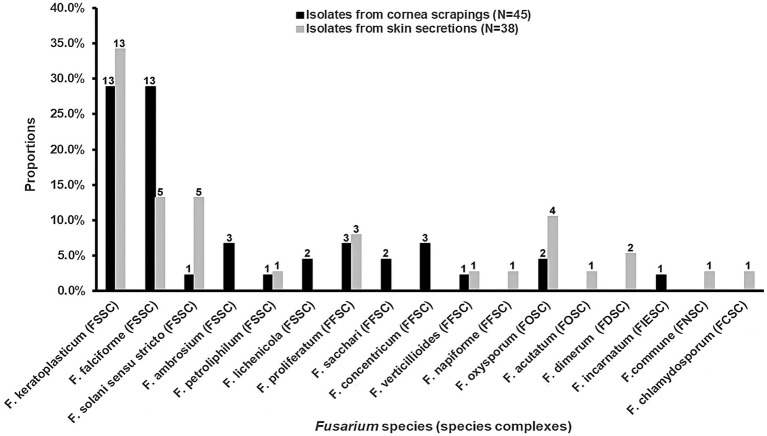
The distributions and proportions of *Fusarium* strains among isolates from cornea scrapings and skin secretions, respectively. FSSC, *F. solani* species complex (SC); FFSC, *F. fujikuroi* SC; FOSC, *F. oxysporum* SC; FDSC, *F. dimerum* SC; FIESC, *F. incarnatum-equiseti* SC; FNSC, *F. nisikadoi* SC; FCSC, *F. chlamydosporum* SC.

For the 45 isolates obtained from cornea scrapings, the detection rates of FSSC, FFSC and FOSC were 73.3% (33/45), 20.0% (9/45) and 4.4% (2/45), respectively. Both *F. keratoplasticum* (28.9%, 13/45) and *F. falciforme* (28.9%, 13/45) within FSSC were the most common species from cornea scrapings (Figure 1). And 63.2% (24/38) of isolates originating from skin secretions belonged to FSSC, followed by FFSC (13.2%, 5/38) and FOSC (13.2%, 5/38). The most prevalent species from skin secretions was *F. keratoplasticum* (34.2%, 13/38; Figure 1).

### MALDI-TOF MS

**Figure 2:**
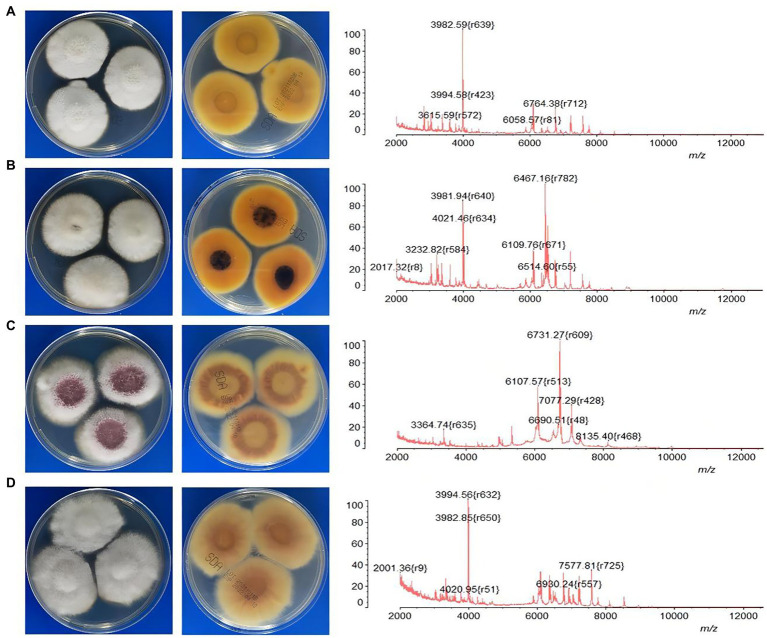
The characteristics of MALDI-TOF MS profiles corresponding to the morphologies of four common *Fusarium* species. **(A)** *F. keratoplasticum*; **(B)** *F. falciforme*; **(C)** *F. proliferatum*; **(D)** *F. oxysporum*.

Comparison of data with DNA sequencing and MALDI-TOF MS is listed in Table 1. The results showed that 91.6% (87/95) of isolates were identified at the SC level by MALDI-TOF MS. For FSSC (*n* = 67) and FDSC (*n* = 2), all the isolates were correctly recognized. Most of isolates were also identified by MALDI-TOF MS for FFSC (75.0%, 12/16) and FOSC (85.7%, 6/7). However, MALDI-TOF MS correctly identified 11.6% (11/95) of the isolates down to the species level, including all isolates of *F. proliferatum* (*n* = 7), *F. verticillioides* (*n* = 2) and *F. dimerum* (*n* = 2). One isolate of *F. concentricum* and two isolates of *F. sacchari* were misidentified as *F. proliferatum* but were correct at the SC level. Further, we analyzed the MALDI-TOF MS profiles of *Fusarium* species corresponding to the morphological characteristics of cultures. Although it was hard to differentiate them by morphology, the discrepancies of MS profile characteristics were observed significantly among these species (Figure 2).

**Figure 3:**
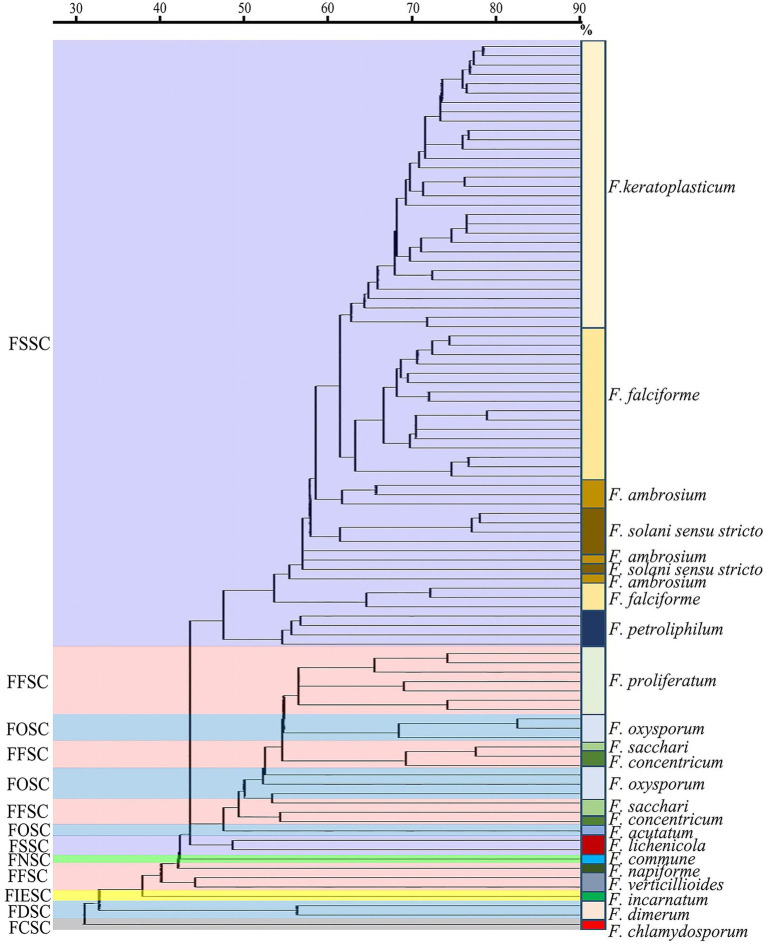
The MALDI-ToF dendrogram of 95 clinical *Fusarium* strains. FSSC, *F. solani* species complex (SC); FFSC, *F. fujikuroi* SC; FOSC, *F. oxysporum* SC; FNSC, *F. nisikadoi* SC; FIESC, *F. incarnatum-equiseti* SC; FDSC, *F. dimerum* SC; FCSC, *F. chlamydosporum* SC.

In the MALDI-TOF dendrogram, almost all of members were found to cluster together in the FSSC except *F. lichenicola* (Figure 3). However, members of FFSC and FOSC were randomly interspersed with those of other species complexes. The strains of the *F. keratoplasticum* within FSSC were found to cluster together in the dendrogram. Differences between *F. proliferatum* and other strains were also unambiguous.

### Antifungal susceptibility

The MICs varied among different species complexes to these antifungal agents (Table 2). Compared to itraconazole and terbinafine, voriconazole and amphotericin B showed lower MICs to most of species. *Fusarium* isolates showed variable MICs to voriconazole ranging between 0.5 and 16 μg/ml. Amphotericin B had good activity against most of species, with 1–16 μg/ml in FSSC, 1–4 μg/ml in FFSC and 1–2 μg/ml in FOSC, respectively. Interestingly, 10.5% (10/95) of strains for amphotericin B had high MICs (≥8 μg/ml), totally belonging to the FSSC. For itraconazole, 93.7% (89/95) of strains showed high MICs (≥32 μg/ml). There were 76.8% (73/95) of strains with high MICs (≥8 μg/ml) for terbinafine. And terbinafine showed low MICs in FFSC (GM = 2.3 μg/ml) and FCSC (1 μg/ml). Compared to the other species complexes, FSSC presented relatively higher MICs to these antifungal agents.

**Table 2:** Activities of antifungal agents against seven *Fusarium* species complexes (SC).

SC (No.)	Antifungal agents MIC (μg/ml)			
	Voriconazole	Itraconazole	Amphotericin B	Terbinafine
***F. solani*** **SC (67)**				
MIC_50_	2	≥32	2	≥32
MIC_90_	8	≥32	8	≥32
MIC range	1–16	≥32	1–16	4–≥32
GM MIC	2.8	32.0	2.9	28.3
***F. fujikuroi*** **SC (16)**				
MIC_50_	2	≥32	1	2
MIC_90_	4	≥32	2	4
MIC range	1–8	2–≥32	1–4	1–4
GM MIC	2.4	19.0	1.5	2.3
***F. oxysporum*** **SC (7)**				
MIC_50_	4	≥32	2	≥32
MIC_90_	8	≥32	2	≥32
MIC range	1–8	4–≥32	1–2	1–≥32
GM MIC	3.0	23.8	1.5	11.9
***F. dimerum*** **SC (2)**				
MIC range	2	≥32	1–2	4–8
***F. chlamydosporum*** **SC (1)**				
MIC	0.5	1	0.5	1
***F. nisikadoi*** **SC (1)**				
MIC	8	≥32	0.25	≥32
***F. incarnatum-equiseti*** **SC (1)**				
MIC	4	≥32	2	≥32

MIC, minimal inhibitory concentration; MIC_50_, the lowest concentration that inhibited the growth of half of the strains; MIC_90_, the lowest concentration that inhibited the growth of 90% of the strains; GM MIC, the geometric mean of MICs.

We further analyzed antifungal activities of species within FSSC (Table 3). The MICs of *Fusarium* isolates to voriconazole ranged from 1 to 16 μg/ml. All strains within FSSC showed high MICs (≥32 μg/ml) for itraconazole. For terbinafine, there were 65.3% (62/95) of strains with highest MICs (≥32 μg/ml). Among the 10 strains with high MICs (≥8 μg/ml) for amphotericin B, nine strains belonged to *F. keratoplasticum* and only one were in *F. falciforme*. Remarkably, high MICs (≥ 32 μg/ml) both for terbinafine and itraconazole were observed among these 10 strains.

**Table 3:** Activities of antifungal agents against different species in *F. solani* species complex.

Species (No.)	Antifungal agents MIC(μg/ml)			
	Voriconazole	Itraconazole	Amphotericin B	Terbinafine
***F. keratoplasticum*** **(31)**				
MIC_50_	2	≥32	4	≥32
MIC_90_	4	≥32	8	≥32
MIC range	1–8	≥32	2–16	≥32
GM MIC	2.6	32.0	4.4	32.0
***F. falciforme*** **(19)**				
MIC_50_	2	≥32	2	≥32
MIC_90_	8	≥32	4	≥32
MIC range	1–8	≥32	1–8	8–≥32
GM MIC	2.4	32.0	2.4	29.7
***F. solani sensu stricto*** **(6)**				
MIC_50_	4	≥32	2	≥32
MIC_90_	4	≥32	2	≥32
MIC range	4	≥32	1–2	8–≥32
GM MIC	4.0	32.0	1.8	25.4
***F. ambrosium*** **(5)**				
MIC_50_	8	≥32	1	8
MIC_90_	16	≥32	1	≥32
MIC range	2–16	≥32	1–2	4–≥32
GM MIC	7.0	32.0	1.3	10.6
***F. petroliphilum*** **(4)**				
MIC_50_	4	≥32	2	≥32
MIC_90_	4	≥32	2	≥32
MIC range	2–4	≥32	1–2	≥32
GM MIC	3.4	32.0	1.7	32.0
***F. lichenicola*** **(2)**				
MIC range	1–2	≥32	2	≥32

MIC, minimal inhibitory concentration; MIC_50_, the lowest concentration that inhibited the growth of half of the strains; MIC_90_, the lowest concentration that inhibited the growth of 90% of the strains; GM MIC, the geometric mean of MICs.

## Discussion

Along with the rising numbers of severely immunocompromised patients in recent decades, invasive or disseminated *Fusarium* infections with high mortality have been found to increase remarkably (Muhammed et al., 2013; Al-Hatmi et al., 2016a). Considering the relatively low susceptibility of *Fusarium* species to most of commonly used antifungal drugs, the prevalence and resistance profile of clinical *Fusarium* species can contribute to enhance the management of the infection (O’Donnell et al., 2008; Guarro, 2013). As a major challenge, it is lack of an accurate, quick and easy to operate approach for the identification of clinical *Fusarium* strains so far. In most of clinical laboratories, *Fusarium* identification mainly depends on different morphological characteristics of size and shape of macro- and microconidia and presence or absence of chlamydospores as well as colony appearance (Najafzadeh et al., 2020; Da et al., 2021). However, a series of factors can affect the morphological characteristics of cultures such as the temperature, the culture medium and maybe the thickness of the medium (Da et al., 2021). *Fusarium* at the SC level are usually hard to be distinguished by this conventional and time-consuming approach if not for experienced experts.

We observed that MALDI-TOF MS had excellent performance of *Fusarium* identification at the SC level with the correct rate up to 91.6% (87/95), taking DNA sequencing of TEF1α as the gold standard (Herkert et al., 2019; Oliveira et al., 2020; Da et al., 2021). Similar results were achieved by Paziani et al. (94.4%) and Song et al. (95.2%; Paziani et al., 2019; Song et al., 2021). To a large extent, it attributed to a success ratio of 100% correct identifications for the most prevalent SC (FSSC; Table 1). High correct rates were also observed for FDSC (100%, 2/2) and FOSC (85.7%, 6/7). For FFSC (*n* = 16), there were four strains unable to be identified by MALDI-TOF MS which were *F. sacchari* (*n* = 1), *F. concentricum* (*n* = 2) and *F. napiforme* (*n* = 1), respectively. Some studies showed good performance of *Fusarium* identification by MALDI-TOF MS down to the species level (Triest et al., 2015; Song et al., 2021). Regrettably, only 11.6% (11/95) of isolates could be correctly identified to the species level in this study. It might be limited by small species and strain representations in commercial libraries (Sleiman et al., 2016). Triest’s study presented a correct rate of the identifications (91.0%) to the species level by constructing an in-house reference spectrum database combined with a standardized MALDI-TOF MS assay (Triest et al., 2015). Song et al. found MALDI-TOF MS recognized 89.04% of *Fusarium* species though a combination of the Bruker library and an expanded version in the BMU database (Song et al., 2021). Further studies will be needed to improve species identification in our laboratory. In the dendrogram, we found all strains except one clustered together in the FSSC, which was similar as Triest’s finding (Triest et al., 2015). However, most of members of the other species complexes were randomly distributed. Normand et al. also demonstrated about 30% of the strains clustered correctly in the dendrograms (Herkert et al., 2019). Given the identification probably depends on recognition of a limited number of conserved proteins regardless of intraspecific variability, phylogenetic interpretation of MALDI-TOF data is not recommended.

The discrepancy of *Fusarium* distribution has been thought to be associated with several factors such as geographical regions, clinical patient populations and infection sites. When being judged from numerous literature data, members of *fusaria* encountered in human infections are mostly found in three species complexes: FSSC, FFSC, and FOSC. FSSC is considered as the most frequently detected SC worldwide, mainly causing superficial infections such as keratitis and onychomycosis under tropical and subtropical climatic conditions, especially in Asia and Latin America (Castro López et al., 2009; Salah et al., 2015; Sun et al., 2015; Guevara-Suarez et al., 2016; Muraosa et al., 2017; Rosa et al., 2017; Tupaki-Sreepurna et al., 2017; Dallé da Rosa et al., 2018; Najafzadeh et al., 2020). Several studies showed FFSC to be the prevalent SC in some areas such as Iran and Turkey, whereas FOSC was more common in Europe (Dalyan Cilo et al., 2015; Abastabar et al., 2018; Oliveira et al., 2019; Najafzadeh et al., 2020; Walther et al., 2021). Our results demonstrated FSSC (70.5%, 67/95) was the most prevalent group mainly originating from corneal scrapings (33/45), followed by FFSC (16.8%, 16/95) and FOSC (7.4%, 7/95). The prevalence of *Fusarium* SC here showed similar as Song’s finding in Northern China and Sun’s finding in central China (Sun et al., 2015; Song et al., 2021).

There were 40.0% (38/95) of isolates in this study that were obtained from skin secretions, a proportion of which were collected from inpatients with burns or diabetes mellitus (data not shown). Severe burns and poorly controlled diabetes are thought to be high risk factors for invasive mold infections (Nucci and Anaissie, 2007; Enoch et al., 2017). However, little is known about the epidemiology of *Fusarium* strains causing locally invasive skin infection in patients with burns or diabetes mellitus, limited by sporadic case reports (Nucci and Anaissie, 2002; Taj-Aldeen et al., 2006; Pai et al., 2010; Atty et al., 2014; Rosanova et al., 2016; Karadag et al., 2020; Tram et al., 2020; Liza et al., 2021). We observed 63.2% (24/38) of isolates from skin secretions belonged to FSSC. Limited by incomplete clinical data here, further studies will be needed to investigate the association of *Fusarium* strains and locally invasive skin infection among these patients. Remarkably, we found one isolate of *F. commune* obtained from skin secretion. *F. commune* within FNSC has been reported as a plant pathogen (Mezzalama et al., 2021; Wang et al., 2022). To the best of our knowledge, this is the first to report this species in clinical specimens.

In Nucci’s review, *F. solani sensu stricto* was regarded as the most common species, followed by *F. oxysporum* and *F. verticillioides* (Nucci and Anaissie, 2007). However, the three most common species were *F. falciforme* and *F. keratoplasticum*, followed by *F. oxysporum* in Al-Hatmi’s review (Al-Hatmi et al., 2016a). Song et al. demonstrated the most prevalent species was *F. solani sensu stricto* (93.8%, 135/144) within the FSSC, and *F. verticillioides* (60.6%, 40/66) within the FFSC (Song et al., 2021). Walther et al. presented *F. petroliphilum* within the FSSC was the most prevalent species (Walther et al., 2021). We here found that 46.3% (31/67) of isolates belonged to *F. keratoplasticum* within the FSSC, followed by *F. falciforme* (28.4%, 19/67) and *F. solani sensu stricto* (9.0%, 6/67). For FFSC, *F. proliferatum* (43.8%, 7/16) was the most common species. Given species-specific differences in antifungal susceptibility, the discrepancy of species distribution should be considered on the treatment options.

Currently, most of *Fusarium* infection still based on empirical antifungal therapy. A limited number of studies on *in vitro* susceptibility were available, showing variable results. In this study, antifungal susceptibility profiles of 95 strains were analyzed for four commonly used agents, i.e., amphotericin B, voriconazole, itraconazole and terbinafine. Our results showed high MICs for itraconazole (93.7%, MIC ≥ 32 μg/ml) and terbinafine (76.8%, MIC ≥ 8 μg/ml) in most of species. Rosa et al. presented higher MICs (≥64 μg/ml) for itraconazole and terbinafine in general (Rosa et al., 2017), while more than 50% of *Fusarium* strains were sensitive to these agents in Sun’s study (Sun et al., 2015). Here, terbinafine showed low MICs in FFSC (GM = 2.3 μg/ml), showing similar results as Song’s study (Song et al., 2021). However, Song et al. presented good activities for terbinafine against FSSC (GM = 2.4 μg/ml) and FOSC (GM = 2.5 μg/ml), which were significantly different from our results (Table 3). For voriconazole, it is thought to be clinically effective against *Fusarium* spp., despite variable *in vitro* activity (Walther et al., 2021). Similarly, the MICs for voriconazole here ranged from 0.5 to 16 μg/ml. Castro López et al. showed *F. solani sensu stricto* had the highest MIC for voriconazole (Castro López et al., 2009). Interestingly, here the MIC of all the *F. solani sensu stricto* strains was 4 μg/ml for voriconazole. In line with our results, several studies showed low MICs for amphotericin B to the majority of isolates (Al-Hatmi et al., 2015b; Rosa et al., 2017; Oliveira et al., 2019, 2020). Remarkably, we observed 10.5% (10/95) of strains with high MICs for amphotericin B (≥8 μg/ml), terbinafine (≥32 μg/ml) and itraconazole (≥32 μg/ml) simultaneously, which were totally belonged to the FSSC. More attentions should be paid on these multi-resistance strains within the FSSC. It is worth noting that information on the relationships between low MIC and clinical response to therapy is still unavailable due to lack of species-specific clinical breakpoints.

Our study has some limitations. Clinical data was not fully collected, preventing us to decipher whether these clinical isolates were related to proven fusariosis or could be associated with contamination of organs. In summary, our results demonstrated that MALDI-TOF MS exhibited good performance on the identification of *Fusarium* strains at the SC level. In most of species, amphotericin B and voriconazole showed lower MICs compared to itraconazole and terbinafine. *F. keratoplasticum* within the FSSC was the most prevalent species in southern China, showing relatively high MICs for these antifungal agents. Further studies will be needed for investigating the correlations of low and high MICs with the prognosis of patients as well as the resistance mechanisms of *Fusarium* strains.

## Data availability statement

The data presented in the study are deposited in the GenBank repository, accession number ON959267–ON959361.

## Author contributions

KL and YP participated in research design and data analysis. PG participated in the writing of the manuscript and data analysis. JC performed the experiments. YT, LX, WZ, XL, YJ, RL, and CC participated in the collection of *Fusarium* strains. All authors contributed to the article and approved the submitted version.

## Conflict of interest

The authors declare that the research was conducted in the absence of any commercial or financial relationships that could be construed as a potential conflict of interest.

## Publisher’s note

All claims expressed in this article are solely those of the authors and do not necessarily represent those of their affiliated organizations, or those of the publisher, the editors and the reviewers. Any product that may be evaluated in this article, or claim that may be made by its manufacturer, is not guaranteed or endorsed by the publisher.

## Footnotes

^1^ https://www.eucast.org/

^2^ http://www.ncbi.nlm.nih.gov/genbank/
